# Molecular Mechanism of Cellular Membranes for Signal Transduction

**DOI:** 10.3390/membranes12080748

**Published:** 2022-07-30

**Authors:** Julia Schumann

**Affiliations:** University Clinic and Outpatient Clinic for Anesthesiology and Operative Intensive Care, University Medicine Halle (Saale), Franzosenweg 1a, 06112 Halle (Saale), Germany; julia.schumann@uk-halle.de

Membranes are central to cell function and crucial to life. Among the essential tasks of biological membranes is to ensure cellular communication processes. Membrane-initiated signal transduction directly regulates enzyme activities, triggers the formation of secondary messengers, and ultimately controls gene expression. Cellular membranes are not rigid, passive structures; rather, they are actively involved in signaling. Membrane composition expresses the levels of channel proteins and receptors, along with the localization of these membrane proteins within membrane microdomains modulating membrane processes and adjusting to the needs of the cell, the surrounding tissue, and the overall organism. This Special Issue covers cutting-edge insights into the molecular mechanisms of signal transduction, with a focus on cellular membranes. A total of five contributions are included, namely three articles and two reviews. In their article Talbi et al. [1] provided insightful data on the regulation of the chloride channel TMEM16A by Ca^2+^/calmodulin (CAM). In TMEM16A-overexpressing HEK293 cells, Ca^2+^ sensitivity of the chloride channel evidently was modulated by interaction with CAM resulting in an increase in sensitivity. This finding explained that overexpressed TMEM16A was active even at basal Ca^2+^ levels. In epithelial cells endogenously expressing TMEM16A, however, no clear impact of CAM was seen. The authors concluded that data from overexpression studies may not be readily extrapolated to the endogenous receptor and physiological stimuli. Hsiao et al. [2] addressed the mode of action of PT-2385, a selective inhibitor of hypoxia-inducible factor 2α (HIF-2α). They were able to show that PT-2385 inhibited the amplitude, gating, and hysteresis of delayed-rectifier K^+^ current (I_K(DR)_) in a time- and concentration-dependent manner. The effects on ionic currents appeared to be non-canonical and upstream of the inhibition of HIF-2α. Galez et al. [3] provided original data concerning the regulation of membrane protein homeostasis. The stability of mRNA transcripts encoding proteins of the plasma membrane was shown to be negatively affected by tethering of the mRNA binding proteins Puf1 and Puf2. Both Puf1 and Puf2 were identified as authentic substrates of the protein kinase Ypk1, which was under the control of TORC2. From this, it was deduced that the TORC2-Ypk1 signaling axis regulated the expression of plasma membrane proteins at the post-transcriptional level. Indeed, phosphorylation of Puf proteins by Ypk1 resulted in increased levels of both mRNA transcripts and associated protein products. Furthermore, this study demonstrated that abrogation of Puf1-mediated mRNA destabilization did not necessarily require the dissociation of phosphorylated Puf1 from the transcript. The two review articles included in this Special Issue provide detailed insight into (i) the role of biological membranes in cancer pathogenesis and (ii) the importance of membrane lipids and lipid translocation within the membrane for cellular signal transduction. In their review, Durán-Saenz et al. [4] reported on interactions of the membranes of cancer cells and platelets. In particular, attention was paid to how platelets can promote tumor growth and support the metastatic process as well as to the role of membrane-membrane interactions in this. Ristovski et al. [5] presented a detailed overview of the function of the ATP-dependent ABC and P4-ATPase lipid transporters. Emphasis was given to the role of these lipid transporters together with membrane lipids, i.e. cholesterol and phospholipids, in cellular signal transduction. Overall, this Special Issue provides state-of-the-art evidence regarding membrane molecular assembly, signal transduction processes affecting membrane composition as well as the ways membranes impact on signaling cascades. Recognized experts in the relevant fields of science reviewed all of the articles published in this Special Issue.

## References

[1] Talbi K., Ousingsawat J., Centeio R., Schreiber R., Kunzelmann K. (2021). Calmodulin-Dependent Regulation of Overexpressed but Not Endogenous TMEM16A Expressed in Airway Epithelial Cells. Membranes.

[2] Hsiao H.-T., Lu G.-L., Liu Y.-C., Wu S.-N. (2021). Effective Perturbations of the Amplitude, Gating, and Hysteresis of *I*_K(DR)_ Caused by PT-2385, an HIF-2α Inhibitor. Membranes.

[3] Galez H.A., Roelants F.M., Palm S.M., Reynaud K.K., Ingolia N.T., Thorner J. (2021). Phosphorylation of mRNA-Binding Proteins Puf1 and Puf2 by TORC2-Activated Protein Kinase Ypk1 Alleviates Their Repressive Effects. Membranes.

[4] Duran-Saenz N.Z., Serrano-Puente A., Gallegos-Flores P.I., Mendoza-Almanza B.D., Esparza-Ibarra E.L., Godina-Gonzalez S., Gonzalez-Curiel I.E., Ayala-Lujan J.L., Hernandez-Barrales M., Cueto-Villalobos C.F. (2022). Platelet Membrane: An Outstanding Factor in Cancer Metastasis. Membranes.

[5] Ristovski M., Farhat D., Bancud S.E.M., Lee J.-Y. (2021). Lipid Transporters Beam Signals from Cell Membranes. Membranes.

